# Higher expression of somatic repair genes in long-lived ant queens than workers

**DOI:** 10.18632/aging.101027

**Published:** 2016-09-06

**Authors:** Eric R. Lucas, Eyal Privman, Laurent Keller

**Affiliations:** ^1^ Department of Ecology and Evolution, Biophore, University of Lausanne, 1015 Lausanne, Switzerland; ^2^ Department of Vector Biology, Liverpool School of Tropical Medicine, Pembroke Place, Liverpool, L3 5QA, UK; ^3^ Department of Evolutionary and Environmental Biology and Institute of Evolution, University of Haifa, 31905 Haifa, Israel

**Keywords:** ageing, lifespan, somatic maintenance, social insects, gene expression

## Abstract

Understanding why organisms senesce is a fundamental question in biology. One common explanation is that senescence results from an increase in macromolecular damage with age. The tremendous variation in lifespan between genetically identical queen and worker ants, ranging over an order of magnitude, provides a unique system to study how investment into processes of somatic maintenance and macromolecular repair influence lifespan. Here we use RNAseq to compare patterns of expression of genes involved in DNA and protein repair of age-matched queens and workers. There was no difference between queens and workers in 1-day-old individuals, but the level of expression of these genes increased with age and this up-regulation was greater in queens than in workers, resulting in significantly queen-biased expression in 2-month-old individuals in both legs and brains. Overall, these differences are consistent with the hypothesis that higher longevity is associated with increased investment into somatic repair.

## INTRODUCTION

Since senescence is a detrimental process with important societal and economic impacts, substantial effort has been invested into understanding its causes and many theories have been proposed to explain its origins [1-9]. One of these theories proposes that senescence is caused by macromolecular damage that accumulates with age due to incomplete somatic maintenance [5,10]. Lifespan is thus expected to be modulated by investment into physiological processes of damage prevention and repair. So far, investigations of somatic maintenance have mostly focused on systems of damage prevention such as anti-oxidants, and have for the most part refuted the hypothesis that longevity is achieved through damage prevention [11-18]. A possible explanation for this patterns is that there is a limited potential to freely modulate the amount of reactive oxygen species because they are important signalling molecules [19,20]. Such constraints are unlikely to apply to systems of macro-molecular repair, which may effectively affect lifespan by modulating the accumulation of damage with age.

Various forms of macromolecular damage have been linked to senescence. For example, DNA may be damaged or mutated in several ways, and there is evidence from mammalian studies that mutations to genes involved in DNA repair accelerate senescence [21]. Similarly, the cellular accumulation of damaged proteins can be toxic and a range of maintenance mechanisms exist to keep this accumulation in check, many of which have been linked to ageing and longevity [22]. One such mechanism is the Ubiquitin Proteasome System (UPS), which degrades mis-folded or damaged proteins by labelling them with ubiquitin and subsequently degrading them. Subunits of the proteasome involved in the UPS have been found to be associated with lifespan and stress resistance in a range of species, from yeast to humans [23-26].

The aim of this study is to investigate whether natural variation in lifespan is associated with differential expression of genes involved in the repair of DNA and proteins. To study the role of these somatic repair genes, we take advantage of the striking variation found in social insects, where queens and workers can differ in their lifespan by more than an order of magnitude [27]. Importantly, the difference in lifespan must be due to differences in gene expression, since there are usually no genetic differences between castes [28,29]. A particularly interesting species for studies of ageing is the ant *Lasius niger*, where queens can survive as long as 29 years [30] whereas workers live for only one or two years even in laboratory conditions [31]. Since lifespan is expected to be modulated by investment into somatic damage repair, we test the prediction that queens of *L. niger* have higher expression of somatic repair genes than workers.

## RESULTS

Twenty somatic repair genes were identified from the literature and mapped to orthologs in the *L. niger* transcriptome. These genes had roles in four DNA repair pathways and one protein maintenance pathway (Supplementary Material Table S1). Two hundred and forty four genes in the transcriptome were annotated with the Gene Ontology (GO) term “DNA repair”, and 162 genes were annotated with the GO term “proteasome-mediated ubiquitin-dependent protein catabolic process” (PUPCP).

**Figure 1:**
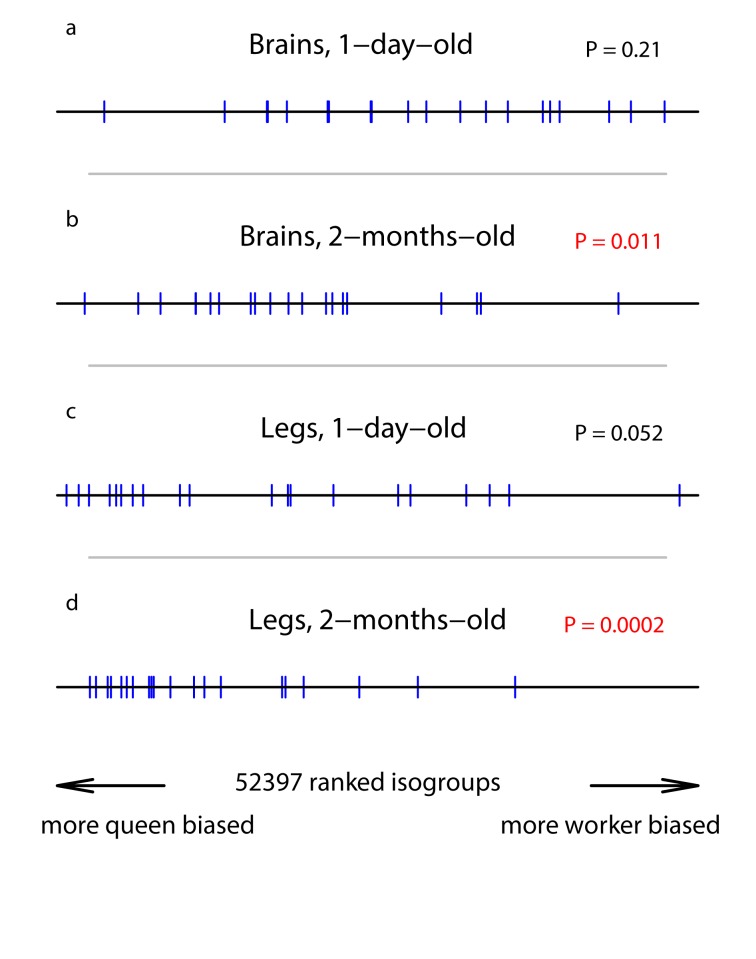
Position of our set of somatic repair genes in a ranked list of all isogroups The horizontal line represents the list of isogroups, ranked according the their significance in bias towards queens or workers, with isogroups in the middle showing relatively unbiased expression. Each vertical bar represents the position of one of our candidate genes. The P-values were generated by the GSEA analysis and represent a test of the null hypothesis that the blue bars are randomly distributes along the black line. (**a**) RNA extracted from brains of 1-day-old individuals. (**b**) RNA extracted from the brains of 2-month-old individuals. (**c**) RNA extracted from legs of 1-day-old individuals. (**d**) RNA extracted from legs of 2-month-old individuals.

In 1-day-old individuals, the level of expression of the 20 somatic repair genes was similar for queens and workers both for the brains and the legs (there was a tendency for higher expression in queens than workers for the legs, but the difference was not significant; Table 1, Figure 1a&c). Over the following two months, the level of expression of the 20 somatic repair genes increased in both castes for both the legs and brains, the increase being significant in all caste/tissue combinations except worker brains (queen brains: GE = 6.7, *n* = 20, *P* = 0.02; worker brains: GE = 4.1, *n* = 20, *P* = 0.34; queen legs: GE = 9.6, *n* = 20, *P* < 0.0002; worker legs: GE = 9.9, *n* = 20, *P* < 0.0002). There was a tendency for a greater increase with age in queens than workers (although non-significantly so; brains: GE = 5.77, *n* = 20, *P* = 0.051; legs: GE = 5.1, *n* = 20, *P* = 0.13), resulting in a significant queen-bias in the expression of the 20 somatic repair genes in both tissues in 2-month-old individuals (Table 1, Figure 1b&d). Expression of these genes was therefore queen-biased in an age-dependent manner, with 2-month-old queens showing significantly higher expression than workers of the same age.

**Table 1:** Results of Gene Set Enrichment Analysis (GSEA) investigating whether the somatic repair gene set was differentially expressed by caste

	1-day	2-month
brains	P=0.21 n=20	P=0.011 (Q) n=20
legs	P=0.052 n=20	P=0.0002 (Q) n=20

P: the P-value of the GSEA analysis; Q/W: indicates whether the bias is towards queens or workers; n: the number of genes involved in the GSEA analysis.

Genes annotated with the GO term “DNA repair” showed no caste-bias in expression in 1-day-old individuals (Table 2). The level of expression of these genes increased over the first two months of adulthood, the increase being significant in all caste/tissue combinations except worker brains (queen brains: *lfdr* = 0.0008; worker brains: *lfdr* = 0.15; queen legs: *lfdr <* 0.0001; worker legs: *lfdr* < 0.0001). This increase in DNA repair gene expression tended to be greater in queens than workers (brains: *lfdr* = 0.083; legs: *lfdr* = 0.15), leading to a significant queen-bias in the expression of genes annotated with the GO term “DNA repair” in the legs of 2-month-old individuals, but not in brains (Table 2).

**Table 2:** Results of enrichment analysis for the GO terms “DNA repair” and “proteasome-mediated ubiquitin-dependent protein catabolic process” (PUPCP)

		1-day	2-month
brains	DNA repair	lfdr=0.11	lfdr=1
	PUPCP	lfdr=0.53	lfdr=0.7
legs	DNA repair	lfdr=0.23	lfdr=0.013 (Q)
	PUPCP	lfdr=0.014 (Q)	lfdr=0.0005 (Q)

lfdr: local fdr values (significance threshold = 0.05); Q/W: indicates whether bias is towards queens or workers

Genes annotated with the GO term PUPCP were more highly expressed in queens than workers in 1-day-old individuals in legs, but not in brains (Table 2). The level of expression of these genes decreased during the first two months of adulthood, though the difference was not significant (queen brains: *lfdr* = 0.22; worker brains: *lfdr* = 0.39; queen legs: *lfdr* = 0.45; worker legs: *lfdr* = 0.36). In 2-month-old individuals, there was no significant difference in the level of expression of genes annotated with the GO term PUPCP in the brains of queens compared to workers (Table 2). By contrast, the level of expression of these genes was significantly higher in queen legs than in worker legs (Table 2).

Overall, our results show a higher level of expression of somatic repair genes in queens than workers, particularly in 2-month-old individuals, in which DNA and protein repair genes were both more highly expressed in queens.

## DISCUSSION

Our analysis of 20 somatic repair genes revealed that queens and workers did not differ in their pattern of expression in 1-day-old individuals. The level of expression of these genes increased with age and this up-regulation was slightly greater in queens than in workers, resulting in significantly queen-biased expression of the 20 somatic repair genes in 2-month-old individuals in both legs and brains. Similarly, analysis of 244 genes annotated with the GO term “DNA repair” revealed no effect of caste on expression in 1-day-old individuals, but a greater up-regulation with age in queens than workers, resulting in significant queen-biased expression in the legs of 2-month-old individuals.

Overall, the combination of these analyses indicates a lack of concerted differences in somatic repair gene expression between 1-day-old queens and workers, but a significantly higher level of expression in queens than workers in 2-month-old individuals. Two previous studies in social insects have compared the expression of somatic repair genes between queens and workers, with contrasting results. In the ant *Harpegnathos saltator*, gamergates (workers that assume the role of the queen after her removal) showed higher expression of Telomerase Reverse Transcriptase than workers [32], but in honeybees, no queen bias was found in the expression of nine DNA repair genes [33]. However, neither of these studies controlled for age, thus confounding its effect with that of caste. Given our finding that the effect of caste on repair gene expression depends on age, it is difficult to draw firm conclusions from these studies. In other species, there is still no consensus concerning the importance of DNA repair activity in determining lifespan. Support for a role of DNA repair in determining lifespan comes from two main sources. First, genetic diseases leading to accelerated ageing in humans are typically associated with defects in the DNA repair pathway [34,35]. Second, inter-specific comparisons of DNA repair activity in mammals have found that longer-lived species tend to display higher DNA repair activity [36-40]. Evidence against the importance of DNA repair in determining lifespan comes from mutant models that disrupt repair pathways but do not show reduction in longevity and may even have extended lifespans [41,42]. Few studies have investigated whether lifespan extension is associated with increased expression of DNA repair genes. Mutant mice over-expressing telomerase or the repair gene MTH-1 show extended lifespan and signs of delayed ageing [43,44], but over-expression of a polymerase important in the repair of DNA damage, PARP-1, had the opposite effect [45]. Similarly, *Drosophila* mutants over-expressing DNA repair genes may either extend or reduce lifespan [46-48]. A limitation of these studies is that expression is manipulated by inserting gene sequences into the genomes of the organisms, affording little control over the resulting degree of over-expression. Such coarse manipulation of a few genes may increase expression to levels above that which is beneficial to the organism or disrupt the balance of essential pathways. Controlling expression in this way may therefore offer only limited information on the importance of a gene in the modulation of lifespan.

The effects of age on the expression of somatic repair genes have been studied in humans, mice, rats, fruit flies and honeybees through targeted studies of specific genes [33,49-53] and through genome-wide expression analyses [54-60]. Overall, these studies provide little consensus on the direction in which somatic repair genes are regulated with age, as differences vary between genes and between studies. However, these studies either investigated individual genes or performed unfocused whole-genome analyses. In contrast, our study focuses specifically on somatic repair genes while performing a single analysis of many genes combined. This provides a more powerful method of investigating the global trends in somatic repair expression changes with age.

The analysis of the 162 genes annotated with the GO term “proteasome-mediated ubiquitin-dependent protein catabolic process” found consistently queen-biased expression in the legs of both 1-day-old and 2-month-old individuals, but no effect of caste on expression in brains at either age. These genes tended to be down-regulated with age in both castes, though non-significantly so. In other species, increased expression of genes involved in the (UPS) has been linked to extended lifespan in yeast [24], worms [26,61], flies [62], rodents [63] and human fibroblasts [64]. Furthermore, in flies, over-expression solely in the neurons was sufficient to extend lifespan [62]. Our findings that queen and worker brains do not differ in the expression of genes involved in the UPS therefore presents an interesting exception to the patterns seen in other species.

Overall, the differences in somatic repair gene expression that we have identified between queens and workers are consistent with the hypothesis that longevity is associated with investment into somatic repair. This contrasts with results from studies investigating the process of damage prevention through anti-oxidant enzymes in social insects, where expression of antioxidant genes was found to be higher in workers than queens, perhaps to compensate for workers' the increased levels of activity [11,16]. Our results suggest that damage repair may be more relevant to lifespan than removal of antioxidants. One reason for this could be the important role that antioxidants play in critical biological processes [19,20], which prevents them from being freely modulated.

## METHODS

### Sample collection

To set up queenless colonies, workers and brood from field colonies were collected from May to June at the UNIL and transferred to controlled climate conditions (25°C, 60% humidity, 12h/12h day/night cycle). These colonies contained pupae, a small number of late-stage larvae and around 100 - 500 workers, but no queen. New queenless colonies were established each year.

Colonies were maintained in a 12h/12h light/dark cycle in controlled climate conditions (25°C, 60% humidity) and fed with a 10% honey solution, mealworms, and an “ant diet” made of agar, eggs, honey and vitamin supplement.

1-day-old and 2-month-old individuals were used. 1-day-old individuals represent a time point at which queens and workers can be considered to have the same biological age, while 2-month-old individuals represent a time point at which queens have successfully founded a colony and are thus in their “typical” state. Older individuals were not included in this analysis because the aim was to detect differences in gene expression that could lead to the accumulation of damage over the course of an individual's life, not only at old age.

Samples were collected in 2012. To obtain 1-day-old queens and workers, queenless colonies were set up as described above and newly emerged queens and workers (identified by the lighter colour of the cuticle) were collected daily and flash-frozen in liquid nitrogen. To obtain 2-month-old workers, worker pupae were transferred from the queenless colonies into queenright colonies and flash frozen in liquid nitrogen two months after emergence. Queenright colonies, established the previous year, were prepared by marking existing workers with paint and removing pupae and large larvae. Each of these colonies then received 40 worker pupae from a queenless colony. The queenright colonies were then checked every 3-4 days for the emergence of workers from the transferred pupae, and an average date between first and last emergence was taken as age 0 for the purposes of age estimation. One week after all the introduced pupae had emerged as workers, the original workers were removed. While retention of paint marks was not complete, original workers in the queenright colonies could be easily distinguished from those of introduced pupae by their small size. In order to make sure that no workers emerged that were not part of the transferred cohort, large larvae and new pupae were regularly removed from the queenright colonies.

To obtain 2-month-old queens, queenright colonies were established in 2012 as described above. The queens were then flash-frozen in liquid nitrogen seven weeks after initial collection (approximately one week after the emergence of the first workers). We assumed the queens to be on average two weeks old on the day of the mating flight, and thus two months old when frozen (as the first queens in laboratory colonies emerged 20 days before the mating flight, it is unlikely that the queen average age estimate was wrong by more than one week).

All samples were frozen within one minute of nest disturbance in order to minimise the effect of disturbance on gene expression. Samples were stored at −80°C.

### Gene expression analysis

#### Tissue preparation

We refer to each of the four age / caste combinations (1-day-old workers, 1-day-old queens, 2-month-old workers and 2-month-old queens) as “treatments”. For all treatments except 2-month-old queens, individuals from different colonies were used for each replicate. 2-month-old queens were collected from the mating flight, making it highly unlikely that any two individuals were from the same colony, so replicates were therefore also independent.

We investigated gene expression in the brain and legs. The brain was chosen because it represents a critical tissue for organismal function and therefore one in which differing levels of somatic repair are likely to be most important. Legs were chosen as a tissue whose function is the same between queens and workers and which should therefore suffer from as little confounding variation as possible.

**Legs**: Six replicates were obtained for each treatment. For each worker replicate, all legs from ten workers were separated from the thorax and pooled. For each queen replicate, all legs from five queens were separated from the thorax and pooled. Leg removals were performed on dry ice.

**Brains**: Five replicates were obtained for each treatment. For each worker replicate, the brains of six workers were dissected and pooled. For each queen replicate, the brains of four queens were dissected and pooled. Dissections were performed in PBS chilled on ice and dissected brains were immediately transferred into TRIZOL and stored at −80°C.

#### RNA extraction

Legs were placed in 1ml Trizol with ceramic beads and homogenised using a shaker (MagNA Lyser, Roche) at 6,000rpm and 4°C. 200μl chloroform was added and the sample mixed by hand and allowed to stand at room temperature for 5 mins. Samples were centrifuged for 15 mins at 13,200rpm and 4°C. The supernatant aqueous phase was removed and added to 500μl isopropanol, mixed by hand and allowed to precipitate at −20°C for 1-2 hrs. Samples were centrifuged for 15 mins at 13,200rpm and 4°C. The supernatant was discarded, the pellets were washed twice with 70% ethanol and re-suspended in 10μl RNAase-free water. DNA was digested in a final concentration of 0.4u/μl DNAase, 1.3u/μl RNAsin, 2mM DTT, 50mM KCl, 5mM MgCl_2 and_ 20mM TRIS (1M), in a final volume of 50μl, incubated for 15mins at 37°C. RNA was then re-extracted by adding 50μl 0.2% SDS and 100μl chloropane, vortexing and centrifuging for 5 mins at room temperature. The aqueous phase was removed and precipitated over-night in 78mM sodium acetate and 70% ethanol at −20°C. After precipitation, samples were centrifuged for 30 mins at 13,200rpm and 4°C. The supernatant was discarded, the pellets were washed twice with 70% ethanol and re-suspended in RNAase-free water. Extractions were stored at −80°C.

RNA was extracted from frozen brains using the same method, except that all volumes were halved before the first RNA precipitation (500μl Trizol, 100μl chloroform, 250μl isopropanol).

#### Library preparation and sequencing

Strand-specific libraries were prepared from the extracted RNA at the Lausanne Genomic Technologies Facility, Center for Integrative Genomics, University of Lausanne, Switzerland using the Illumina TruSeq Stranded mRNA reagent kit (Illumina, San Diego, CA). Samples were barcoded at the library preparation stage for multiplexing. Sequencing was performed by Illumina HiSeq 2000/2500 in 100 nucleotide paired-end mode. Fastq files were produced from the raw data using v1.82 of the Illumina Analysis software. The raw reads obtained from the sequencing have been deposited in the NCBI Short Read Archive (accession number: SRP069113).

**Legs**: The 24 libraries were divided into two groups of 12 (three from each of the four age / caste combinations). The libraries within each group were sequenced together on two lanes of the Illumina platform.

**Brains**: The 20 libraries were sequenced together on four lanes of the Illumina platform. Three of these lanes were revealed to be under-loaded and the libraries were therefore sequenced on a further three lanes. Data from all seven lanes were combined in the analysis.

#### Transcriptome Assembly

We also assembled a draft sequence of the *L. niger* transcriptome from the Illumina RNA sequencing described above. Data from one replicate of each 1-day-old and 2-month-old tissue / caste combination were pooled. Reads were trimmed for low quality and adapter contamination using *Trimmomatic* (v0.30; [65]) and filtered for reads that failed Illumina's quality checks (labelled 'Y' in the read name). The remaining reads were assembled using the software Trinity (release r-2013-02-25; [66]) with a minimum k-mer value of 2 and default values for other variables. Trinity automatically groups assembled sequences into components and sub-components. The contigs within a given sub-component are putative alternative transcript isoforms, and a sub-component is representative of a gene. We will refer to the sub-components as “genes”, but we note that they include non-protein coding sequences.

#### Identifying somatic repair genes

We refer to the processing of damaged DNA and protein, whether through direct repair or through degradation of damaged molecules, as “somatic repair”. Genes with documented roles in a range of somatic repair pathways were identified from the literature (see Supplementary Table S1 for details). Homologs of these genes were then searched for in our transcriptome using the principle of reciprocal top BLAST hits: a given contig in the transcriptome was accepted as a homolog for the focal gene if it was the top translated BLAST hit (tblastn; [67]) of the human copy of this protein against the transcriptome, and the human protein was the top BLAST hit (blastx) of the contig against the human complete Swissprot proteome. All members of that contig's isogroup (i.e.: all of its putative isoforms) were labelled as the same gene.

#### GO term annotation

We assigned Gene Ontology (GO; [68]) terms to all contigs in our transcriptome that had Open Reading Frames (ORF) and showed sufficient homology to known protein sequences based on their top blast hit. ORFs were predicted with Augustus (v2.5.5; [69]) using the honeybee as a model for gene structure. For each gene in the transcriptome, we kept the longest ORF from among the alternative transcripts (isogroup). These were then BLASTed against the UniProt data set with an e-value cut-off of 10E-4. The top BLAST hit for each genes was used to assign GO terms to the corresponding *L. niger* gene using the Blast2Go pipeline (v2.5.0; [70]).

#### Differential gene expression

Raw reads were aligned to the transcriptome using Bowtie2 (v2.1.0; [71]) with default parameter values. Counts of aligned reads were analysed using the R package *edgeR* [72] as follows. Counts of alignments to all transcripts within an isogroup were combined to provide a single count per gene. Normalisation was carried out using Trimmed Mean of M-values [73] and dispersion was calculated by combining trended and tag-wise estimates. *P*-values were obtained using gene-ralised linear modelling implemented in *edgeR*, where caste and age were defined as categorical fixed effects.

#### Gene Set Enrichments Analysis of somatic repair genes

Gene Set Enrichment Analysis (GSEA; [74]) was performed to determine whether our set of identified somatic repair genes globally showed biased expression towards either caste within an age group. To create the ranked list of genes necessary for this approach, all *N* genes in the transcriptome were ranked according to their statistical support for expression bias using a signed *P*-value calculated as c(1-*P*), where c is the sign of the coefficient representing the effect of caste in the model (c = −1 for queen-biased genes and 1 for worker-biased genes). This placed queen-biased contigs with low *P*-values at one end of the rankings, worker-biased contigs with low *P*-values at the other end and contigs with *P*-values close to 1 in the middle. A running sum was then calculated by taking genes in rank order and increasing the value of the sum by 1 if the gene was in our gene set of interest and decreasing it by *n*/(*N*-*n*) otherwise (where *n* is the number of genes in our gene set). The value of the running sum after all genes have been scored is therefore 0, and the Enrichment Score (ES) is calculated as the maximum of the absolute value of the running sum. A null distribution for the ES was obtained by 5000 iterations of re-sampling: *n* genes were randomly sampled from the transcriptome to form a new gene set and the ES was recalculated.

The same method was used to determine whether global expression of the somatic repair genes differed with age, with the genes being ranked according to statistical support for age-bias instead of caste-bias. The same method was also used to determine whether expression of the somatic repair genes showed an interaction between age and caste, with the genes being ranked according to statistical support for the interaction.

#### GO term enrichment analysis

To investigate whether the GO terms for “DNA repair” or “proteasome-mediated ubiquitin-dependent protein catabolic process” showed biased expression with respect to age or caste, local false discovery rate (lfdr) was calculated for all GO terms using genes ranked as described above for the GSEA. The lfdr is in effect the posterior probability, given the observed *P*-value, that the GO term is not biased [75]. We considered a GO term to be enriched for differentially expressed genes if lfdr ≤ 0.05. Analyses were performed in R, using the packages *topGO* [76] to obtain initial *P*-values, and *fdrtool* [77] to calculate lfdr. The *topGO* package was slightly modified to allow the use of a two-tailed Kolmogorov-Smirnov test, which is better suited to the signed *P*-value statistic.

## SUPPLEMENTARY DATA TABLE


