# Supplementary material for: Higher expression of somatic repair genes in long-lived ant queens than workers
Source: Aging (Albany NY). 2016 Sep 6;8(9):1940–9. doi: 10.18632/aging.101027 (PMC5076446; doi:10.18632/aging.101027)
Supplement: Supplementary file 1 [file aging-08-1940-s001.pdf]

SUPPLEMENTARY DATA

Table S1. List of somatic repair genes included in the Gene Set Enrichment Analysis

Gene name	Somatic repair pathway	Uniprot reference number of human gene	Reference	Direction of trend in 1-day-old legs (caps indicate significant bias)	Direction of trend in 2-month-old legs (caps indicate significant bias)	Direction of trend in 1-day-old brains (caps indicate significant bias)	Direction of trend in 2-month-old brains (caps indicate significant bias)
AAG	BER	sp|P29372	Larsen et al 2005	WORKER (P=0)	queen (P=0.06)	worker (P=0.2)	worker (P=0.65)
AlkB8	DR	sp|Q96BT7	Fu et al 2010	queen (P=0.58)	queen (P=0.16)	worker (P=0.76)	queen (P=0.4)
APE1	BER	sp|P27695	Larsen et al 2005	QUEEN (P=0.01)	QUEEN (P=0)	worker (P=0.48)	queen (P=0.85)
EXO1	MMR	sp|Q9UQ84	Larsen et al 2005	queen (P=0.11)	queen (P=0.17)	queen (P=0.2)	queen (P=0.15)
Ku70	NHEJ	sp|P12956	Lombard et al 2005	queen (P=0.87)	worker (P=0.73)	queen (P=0.66)	worker (P=0.83)
Ku80	NHEJ	sp|P13010	Lombard et al 2005	QUEEN (P=0.02)	QUEEN (P=0.01)	queen (P=0.4)	queen (P=0.37)
lig1	BER	sp|P18858	Sancar et al 2004, Waterworth et al 2009	queen (P=0.66)	queen (P=0.27)	worker (P=0.13)	queen (P=0.14)
MGMT	DR	sp|P16455	Larsen et al 2005	worker (P=0.6)	queen (P=0.69)	queen (P=0.99)	queen (P=0.12)
MLH1	MMR	sp|P40692	Larsen et al 2005	QUEEN (P=0.01)	QUEEN (P=0.01)	queen (P=0.65)	queen (P=0.58)
MSH2	MMR	sp|P43246	Larsen et al 2005	worker (P=0.47)	queen (P=0.65)	worker (P=0.15)	queen (P=0.85)
MSH6	MMR	sp|P52701	Larsen et al 2005	QUEEN (P=0)	queen (P=0.07)	queen (P=0.22)	queen (P=0.86)
NTH1	BER	sp|P78549	Larsen et al 2005	QUEEN (P=0)	QUEEN (P=0)	queen (P=0.26)	queen (P=0.63)
OGG1	BER	sp|O15527	Larsen et al 2005	worker (P=0.85)	QUEEN (P=0.03)	queen (P=0.72)	queen (P=0.79)
PMS2	MMR	sp|P54278	Larsen et al 2005	QUEEN (P=0.01)	QUEEN (P=0.04)	worker (P=0.52)	queen (P=0.58)
Rpn11	PU	sp|O00487	Tonoki et al 2009	queen (P=0.52)	queen (P=0.29)	worker (P=0.64)	worker (P=0.64)
Rpn6	PU	sp|O00231	Vilchez et al 2012	queen (P=0.05)	QUEEN (P=0)	queen (P=0.73)	worker (P=0.13)
TDG	BER	sp|Q13569	Larsen et al 2005	queen (P=0.24)	worker (P=0.97)	QUEEN (P=0.01)	queen (P=0.63)
XPA	NER	sp|P23025	Lombard et al 2005	queen (P=0.17)	queen (P=0.1)	queen (P=0.93)	queen (P=0.39)
XPC	NER	sp|Q01831	Lombard et al 2005	worker (P=0.42)	queen (P=0.77)	worker (P=0.89)	QUEEN (P=0.04)
XPF	NER	sp|Q92889	Lombard et al 2005	worker (P=0.79)	worker (P=0.18)	worker (P=0.37)	queen (P=0.55)

Abbreviations:

BER	Base Excision Repair
DR	Direct Reversal
MMR	Mis-Match Repair
NER	Nuceotide Excision Repair
PU	Protein Polyubiquitination
NHEJ	Non-Homologous End Joining

REFERENCES

1. Larsen NB; Rasmussen M, Rasmussen LJ. Nuclear and mitochondrial DNA repair: similar pathways? Mitochondrion. 2005; 5:89-108.
2. Lombard DB, Chua KF, Mostoslavsky R, Franco S, Gostissa M, Alt FW. DNA repair, genome stability, and aging. Cell. 2005; 120:497-512.
3. Fu D, Brophy JAN, Chan CTY, Atmore KA, Begley U, Paules RS, Dedon PC, Begley TJ, Samson LD. Human AlkB homolog ABH8 is a tRNA methyltransferase required for wobble uridine modification and DNA damage survival. Molecular and cellular biology. 2010; 30:2449-2459.
4. Sancar A, Lindsey-Boltz LA, Ünsal-Kaçmaz K, Linn S. Molecular mechanisms of mammalian DNA repair and the DNA damage checkpoints. Annual review of biochemistry. 2004; 73:39-85.
5. Tonoki A, Kuranaga E, Tomioka T, Hamazaki J, Murata S, Tanaka K, Miura M. Genetic Evidence Linking Age-Dependent Attenuation of the 26S Proteasome with the Aging Process. Molecular and Cellular Biology. 2009; 29:1095-1106.

6. Vilchez D, Morantte I, Liu Z, Douglas PM, Merkwirth C, Rodrigues APC, Manning G, Dillin A. RPN-6 determines C. elegans longevity under proteotoxic stress conditions. Nature. 2012; 489:263-270.
7. Waterworth WM, Kozak J, Provost CM, Bray CM, Angelis KJ, West CE. DNA ligase 1 deficient plants display severe growth defects and delayed repair of both DNA single and double strand breaks. BMC plant biology. 2009; 9:79.
